# Why both sides of the gender equation matter

**DOI:** 10.7554/eLife.78890

**Published:** 2022-04-04

**Authors:** Lindy E Barrett

**Affiliations:** 1 Stanley Center for Psychiatric Research, Broad Institute of MIT and Harvard Cambridge United States; 2 Department of Stem Cell and Regenerative Biology, Harvard University Cambridge United States

**Keywords:** gender, inequalities, STEMM

## Abstract

Despite efforts to increase gender diversity in science, technology, engineering, mathematics and medicine (STEMM), men continue to hold most tenured and leadership positions. Moreover, the specific population shifts and timelines which may be required to achieve gender parity have not been well delineated. It is obvious that if women are statistically underrepresented in a field, then men must be statistically overrepresented: however, male overrepresentation and related gender-based advantages are rarely mentioned in conversations about gender equality. It is important that actions to address both overrepresentation and underrepresentation are elements of any strategy that seeks to move STEMM fields closer to gender parity.

## Evaluating gender parity in US academic medicine

Gender differences in academia are deeply rooted and take many forms – unequal pay, inequalities in promotion, sexual harassment, and disparities in the number of men and women in senior leadership positions. For example, studies have reported gender bias in the citation of scientific papers (Larivière et al., 2013), in assessments of the scientific quality of abstracts submitted to conferences (Knobloch-Westerwick et al., 2013), in the language used for letters of recommendation (Trix and Psenka, 2003) and in teaching evaluations (MacNell et al., 2015).

Although the number of women accessing higher education has tripled globally in the last 30 years, there are still more men than women in academic positions in science and medicine (Hurtado, 2021; UNESCO International Institute for Higher Education in Latin America and the Caribbean, 2021). Gender disparities at tenured and leadership levels are particularly high, despite women and men receiving many advanced degrees, such as MDs and PhDs in the biomedical sciences, in roughly equal numbers (Beeler et al., 2019; Llorens et al., 2021; Salinas and Bagni, 2017). For example, as of 2018, only 25% of full professors and 19% of department chairs in academic medicine in the United States (US) were women, as opposed to 75% and 81% of men in these positions, respectively (AAMC, 2020). These ratios roughly parallel those of NIH staff researchers with tenured positions (23% women, 77% men) and tenured faculty in the European Union (21% women, 79% men) (NIH Gender Inequality Task Force Report, 2016; Salinas and Bagni, 2017). It should be noted that these numbers do not include gender-non-conforming individuals.

**Figure 1.**
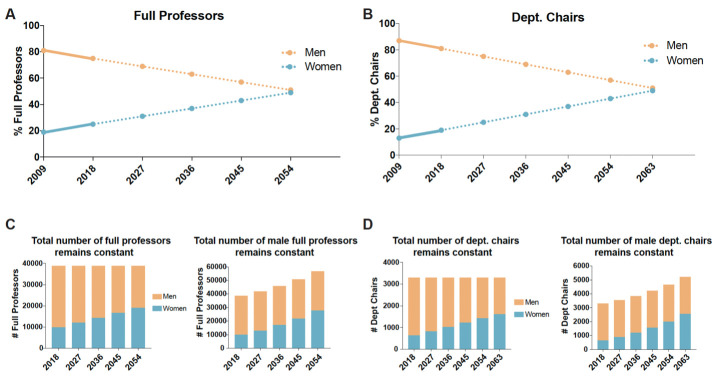
What is needed to achieve gender parity at the full professor and department chair levels in US medical schools? (**A**) The graph shows the percentage of male full professors (orange circles) and female full professors (blue circles) by year. Data from 2009 to 2018 were collected by the AAMC (solid lines), while data from 2018 to 2054 are linear projections (dotted lines) based on the 2009–2018 trends. From these projections, gender parity (within one percentage point) would be achieved in 2054. (**B**) The graph shows the percentage of male department chairs (orange circles) and female department chairs (blue circles) by year. Data from 2009 to 2018 were collected by the AAMC (solid lines), while data from 2018 to 2063 are linear projections (dotted lines) based on the 2009–2018 trends. From these projections, gender parity (within one percentage point) would be achieved in 2063. (**C**) Projections in absolute numbers assuming the total number of full professors remains constant (*left*), or the total number of male full professors remains constant (*right*). To maintain 38,767 full professor positions (the number in 2018, including 23 with gender unreported), the number of women would need to increase by approximately 9,590 (from 9,794–19,384) and the number of men would need to decrease by approximately 9,566 (from 28,950–19,384). Alternatively, if the number of male full professors remains constant, an additional 19,156 full professor positions would be needed to achieve parity. (**D**) Projections in absolute numbers assuming the total number of department chairs remains constant (*left*), or the total number of male department chairs remains constant (*right*). To maintain 3,292 department chair positions (the number in 2018), the number of women would need to increase by 1,007 (from 639 to 1,646) and the number of men would need to decrease by 1,007 (from 2,653–1,646). Alternatively, if the number of male department chairs remains constant, an additional 2,014 department chair positions would be needed to achieve parity. In all panels, data for men is shown in orange and data for women in shown in blue. Data from AAMC, 2020.

Although gender disparities have become smaller over the last decade, progress has been slow: indeed, if the rate of progress over the last decade is maintained, we will not reach gender parity at the full professor level in academic medicine in the US until 2054, and it will be 2063 before there is parity at the department chair level (Figure 1A and B). This slow rate of progress would also fall well short of the pledge made by 80 world leaders at a United Nations summit in 2015 to end gender discrimination by 2030, including the commitment of “reaching parity for women at all levels of decision-making” (UN Women, 2015; UN Women, 2016).

There are essentially two paths to achieving parity: increasing the number of women in these positions while reducing the number of men, or increasing the number of women without reducing the number of men. In 2018, three-quarters of the 38,767 full professors in US medical schools were men. If the total number of full professor positions remains constant, the fraction of women in these positions needs to double, and the fraction of men needs to be reduced by one third, to achieve gender parity (Figure 1C). Alternatively, if the total number of full professor positions is increased to allow the number of female professors to be doubled, without changing the number of male professors, an additional 19,156 new positions will be needed to achieve gender parity (Figure 1C). This latter scenario would require a substantial increase in overall resources and would contrast with trends from the last 30 years showing that the number of faculty positions in science and engineering fields has essentially remained constant (Schillebeeckx et al., 2013).

For department chair positions (which numbered 3,292 in 2018), if the total number remains constant, the number of women needs to increase by a factor of 2.5 (from 639 to 1,646) to reach parity; this would involve just over one thousand male department chairs being replaced by women (Figure 1D). Alternatively, achieving parity by increasing the number of positions would require 2,014 new positions (Figure 1D). This latter scenario would likely require a new model of departmental leadership, such as a system with co-chairs of departments or rotating department chairs. While the situation is better at the assistant professor level, where 54% of posts are held by men and 46% by women, the substantial disparities found at higher levels suggests a need for policies to improve the retention of female professors and ensure that the promotion system does not advantage men or disadvantage women.

It is important to note that gender disparity has many causes, and that simply increasing the number of women in leadership is unlikely to be sufficient to achieve parity. As the number of women in tenured and leadership positions increases, the number of men holding these positions will need to decrease, or alternative models of power and leadership will need to be implemented. Moreover, circumstances unique to each institution or field, such as the early-career pipeline, promotion timelines and retention rates of women and men, will need to be considered to ensure gender parity throughout the career stages.

## Rethinking the reference point

Conversations about inequalities in STEMM fields often use men as the reference point, highlighting the underrepresentation and disadvantage of women relative to men. While this generally implies male overrepresentation and advantage, these issues are frequently overlooked. If one were to ask a group of women in STEMM fields to discuss gender-based disadvantages, one would likely hear numerous examples of career obstacles and instances of bias, discrimination and/or harassment faced by women. By contrast, discussions around gender-based advantages for men at each career stage are far less common. Focusing solely on the underrepresentation and disadvantage of women may contribute to the illusion that men are simply a neutral reference point.

In a highly competitive system, the disadvantages of one group advantage others and vice versa. In STEMM fields, men benefit both from fewer of the disadvantages faced by women and from direct advantages. For example, men experience lower rates of sexual harassment than women (National Academies of Sciences, Engineering, and Medicine, 2018). Moreover, identical curriculum vitae have been reviewed more favorably (by both male and female science faculty) and considered for higher starting salaries when the applicant was thought to be a man, suggesting that men are also given more credit than women for identical achievements (Moss-Racusin et al., 2012).

While men should not be faulted for advantages that are rooted in a historical system, it should no longer be acceptable to ignore the existence of gender-based advantages. Studies have shown that even small advantages or disadvantages can have large cumulative effects over time. As one workplace simulation demonstrated, valuing the performance of a male employee at just 3% above the performance of a female employee resulted in a shorter trajectory for the male employee to reach an executive level, with half the number of successful projects required. This was sufficient to generate substantial male overrepresentation and female underrepresentation at executive levels (Du et al., 2021; Nordell, 2021). Thus, acknowledging and addressing both sides of representation and bias is critical for making strides toward gender parity in academia.

## External advantages may be concealed by attributional biases

Tackling gender disparities in STEMM fields will also require more explicit assessment of advantage in general, and how individuals perceive advantage. In the field of social psychology, attribution theory addresses how we assign causation to life events, which may be internal or external. Biases in the way people attribute causation are well-studied, and our attributions can vary depending on whether we are making them about ourselves or others, or whether they are positive or negative life events.

The self-serving bias refers to the tendency to attribute one’s own success to internal factors and one’s failures to external ones (Miller and Ross, 1975; Wang et al., 2017). In other words, we tend to believe that positive outcomes in life are the product of our own ingenuity or hard work, while negative events in life are due to situation. While this bias is considered to be an important component of adaptive function and mental health, it may also cause people to perceive themselves being better than others, and negatively impact negotiation processes (Babcock and Loewenstein, 1997; Gelfand et al., 2002). It is quite reasonable to think of efforts toward gender equality as part of a broad negotiation, in which individuals have to consider the position of each gender and reach conclusions about what is fair.

**Figure 2.**
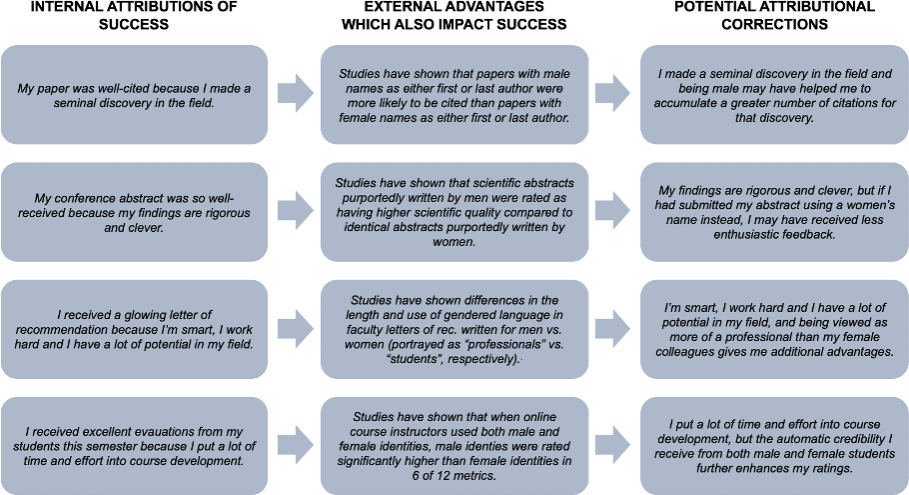
Examples of the self-serving bias and its relevance for gender equality in STEMM fields. Focusing on the self-serving bias, four theoretical internal attributions of success are shown on the left; the corresponding documented external factors, which also impact success, are shown in the middle; and potential attributional corrections are shown on the right. Here, examples were selected from studies of gender bias in paper citations (Larivière et al., 2013), assessment of scientific quality in conference abstracts (Knobloch-Westerwick et al., 2013), faculty letters of recommendation (Trix and Psenka, 2003) and teaching evaluations (MacNell et al., 2015).

Holding the belief that one’s own success in a STEMM field is primarily or entirely driven by ingenuity and arduous work, while ignoring the external factors contributing to success – which in the case of gender, are unequally distributed – is an example of the self-serving bias (Figure 2). Similar to the myth of meritocracy, the self-serving bias may influence perceptions of advantage and could be used to justify current gender disparities and resist equality initiatives, if not corrected. When the American Association of Medical Colleges (AAMC) examined perceptions of equality they found that 85% of men surveyed thought their institute “offered equal opportunities to faculty regardless of gender”, which raises the question of what factors the respondents attributed to the significant gender disparities at their institutes (AAMC, 2020).

Reductions in long-standing advantages, such as shorter promotion timelines or receiving more credit for equal work, could also be mistaken for disadvantages if they are not acknowledged in the first place. Analyses of experiences and perceptions of participants in the ‘Athena SWAN Charter for Women in Science’ reported resentment of what mostly male respondents viewed as positive discrimination – that women were suddenly receiving external advantages, such as faster promotion timelines or new positions created specifically for women, and that this was both unfair and anathema to equality (Ovseiko et al., 2017). Here, it would be extremely informative to know whether respondents perceived themselves as having received external advantages that contributed to their own career successes.

While the above data are not quantitative, they underscore the relevance of further assessing how individuals assign causation to career success and disparities across genders, and how underlying attributional biases may be at play in these analyses. Acknowledging the many external factors accelerating academic career success is particularly important, given their unequal distribution between genders (Figure 2).

## Moving toward gender parity in STEMM fields

Several strategies could be considered to achieve or accelerate gender parity. First, both the current status of gender parity and future projections toward it must be made available for every university, field of research and career stage. This will enable us to understand the full extent of disparity at each level, to analyze how various initiatives are faring, and to clearly recognize when particular institutes are not making progress. The Initiative on Women in Science and Engineering from the New York Stem Cell Foundation piloted a report card for gender equality, which evaluates an institute’s commitment to promoting gender equality (Beeler et al., 2019). Templates like these could be leveraged by funding organizations and professional societies to facilitate data collection and analyses.

Institutes and fields should be explicitly discussing timelines for achieving gender parity, and the specific underlying population shifts that may be required. Does US academic medicine accept a 30- to 40- year timeline to achieve gender parity at tenured and leadership levels? What would be necessary to sustain or accelerate progress given the more equal gender ratios at the assistant professor level? Do various projected population shifts make people uncomfortable, and if so, why? Alternatively, what might unconventional power structures in academia look like and how would they be tested and implemented?

Concepts of male advantage and overrepresentation also must be addressed. To explicitly highlight the position of men in the gender equality equation, it may be worthwhile to consider women as the reference point, rather than men (i.e., men are overrepresented and advantaged relative to women). Practically speaking, the STEMM community’s understanding of male advantage may influence the strategies used to address gender equality, such as giving women explicit advantages versus removing known obstacles.

More research is needed to fully understand how attributional biases may impact perceptions of gender equality in STEMM fields and what we could do to avoid bias. One study of settlement negotiations in a mock trial showed that educating participants about self-serving bias and requiring them to delineate weaknesses in their own position had a positive impact on the negotiation process, suggesting there is room to intercede (Babcock and Loewenstein, 1997).

Finally, when it comes to performing the work required for gender equality initiatives, such as committee activities, data collection and documentation, men are proportionally far less burdened than women (Caffrey et al., 2016). This may further disadvantage women with workloads that are not part of their career advancement and reinforce the perception that gender inequality is an issue that only impacts women and therefore needs to be solved by women. Would men be more likely to take notice if issues were framed from the male perspective? Should business or scientific arguments be made more prominently in addition to moral arguments? Imagine a scenario in which everyone whose last name started with the letters A-M was arbitrarily assigned a higher value, combined with additional obstacles, harassment and discrimination for the latter half of the alphabet sufficient to drive a 50% reduction in their ranks. This would not only be a crisis of fairness and hypocrisy in fields meant to be anchored in truth and objectivity. It would also be a crisis of innovation, insight and progress, which the entire alphabet would be expected to help rectify. Everyone needs to take responsibility for the future of STEMM fields.
